# Adult Children’s Migration and Health-Related Quality of Life Among Older Nepalese Adults

**DOI:** 10.1093/geroni/igaa057.305

**Published:** 2020-12-16

**Authors:** Ghimire Ghimire, Devendra Singh, Sara McLaughlin, Dhirendra Nath, Hannah McCarren, Janardan Subedi, Saruna Ghimire

**Affiliations:** 1 Miami University, Oxford, Ohio, United States; 2 Asian College for Advanced Studies, Lalitpur, Nepal; 3 Grande International Hospital, Kathmandu, Nepal; 4 Miami University, Oxford, United States; 5 Miami University, Miami University, Ohio, United States

## Abstract

Traditionally, adult children have served as primary caretakers and providers for older Nepalese adults. However, out-migration of adult children for employment and other opportunities is increasing. Health-related quality of life (HRQOL) in older Nepalese adults in general and in the context of adult children’s migration is poorly understood. This study aims to assess HRQOL of older Nepali adults and its relationship with adult children’s migration. We used existing cross-sectional survey data on 260 older adults from the Krishnapur municipality, which has witnessed a high rate of adult migration. HRQOL was assessed using the SF-12, which provides a physical (PCS) and mental (MCS) health component. Scores for PCS and MCS range from 0-100; a higher score indicates better HRQOL. Simple and multiple linear regression were used to assess correlates of HRQOL. Participants had suboptimal HRQOL [mean (±SD): PCS =40.4±9.2 and MCS=45.2±7.7]. After adjusting for covariates, adult children’s migration was associated with lower MCS scores (β: -2.33, 95%CI: -4.21, -0.44). Individuals with more than one child had higher MCS scores (β: 2.14, 95%CI: 0.19, 4.09). Females (β: -3.64, 95%CI: -7.21, -0.06) and those with a history of unemployment (β: -6.36, 95%CI: -10.57, -2.15) had lower PCS scores than their respective counterparts. The presence of one or more chronic conditions was associated with significantly lower PCS and MCS. Our findings suggest that out-migration of adult children may negatively effect HRQOL among older Nepali adults, specifically their psychological well-being. Additional research is needed to investigate potential moderating factors that may serve as important buffers.

